# A generalized target theory and its applications

**DOI:** 10.1038/srep14568

**Published:** 2015-09-28

**Authors:** Lei Zhao, Dong Mi, Bei Hu, Yeqing Sun

**Affiliations:** 1 College of Environmental Science and Engineering, Dalian Maritime University, Dalian, Liaoning, P.R. China; 2 Institute of Environmental Systems Biology, Dalian Maritime University, Dalian, Liaoning, P.R. China; 3 Department of Physics, Dalian Maritime University, Dalian, Liaoning, P.R. China

## Abstract

Different radiobiological models have been proposed to estimate the cell-killing effects, which are very important in radiotherapy and radiation risk assessment. However, most applied models have their own scopes of application. In this work, by generalizing the relationship between “hit” and “survival” in traditional target theory with Yager negation operator in Fuzzy mathematics, we propose a generalized target model of radiation-induced cell inactivation that takes into account both cellular repair effects and indirect effects of radiation. The simulation results of the model and the rethinking of “the number of targets in a cell” and “the number of hits per target” suggest that it is only necessary to investigate the generalized single-hit single-target (GSHST) in the present theoretical frame. Analysis shows that the GSHST model can be reduced to the linear quadratic model and multitarget model in the low-dose and high-dose regions, respectively. The fitting results show that the GSHST model agrees well with the usual experimental observations. In addition, the present model can be used to effectively predict cellular repair capacity, radiosensitivity, target size, especially the biologically effective dose for the treatment planning in clinical applications.

Instead of the conventional adopted γ or X rays, high energy charged particles are being widely used in cancer therapy^1^,^2^. The main advantages over γ or X rays are due to the particular physical and radiobiological properties of charged particle radiation, especially, giving rise to a sharp maximum in ionization near the end of the range (Bragg peak) and an enhanced relative biological effectiveness (RBE) (for heavy ions only)^3^,^4^. In the actual radiotherapy, the spread out Bragg peak (SOBP) is used to cover a well defined target volume at a given depth by modulating single peaks^5^.

Radiotherapy is based on the mechanism of radiation-induced cell killing^1^. At the molecular level, it is generally considered that cell killing has been mainly attributed to the radiation energy deposition in the DNA within the nucleus, with production of DNA double-strand breaks (DSB)^1^,^6^. Hence, the hypothesis is raised that the nuclear DNA is the critical target to induce lethal effects as a result of radiation exposure, which is termed “targeted effects”^7^,^8^,^9^. In addition, some new radiobiological experiments show that, there exist “non-targeted effects”^10^, such as bystander effect^11^,^12^, adaptive response^13^,^14^, low dose hyper-radiosensitivity^15^, etc, which do not depend on the amount of energy deposited in DNA.

Different radiobiological models, including “targeted” and “non-targeted” effects models, have been proposed for RBE estimation of radiotherapy, and even risk assessment of space radiation^16^. Lea’s target theory is one of the earliest interpretive models for radiation-induced cell killing^17^. In this theory, the hit probability *p*_0_ for *N* targets to be hit *n* times followed Poisson distribution, which can be expressed as


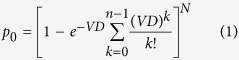


where, *D* represents the radiation dose, and *V* denotes the target volume. When both *N* and *n* are equal to 1, the survival fraction *S* can be reduced to single-hit single-target (SHST) model,
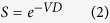


The major drawback of target theory is that it can’t describe the induction of damage by radiation and cellular repair effects^17^,^18^. Only two states in the theory are distinguished, namely, the undamaged surviving and the damaged death. Nevertheless, the target models still have a certain application in radiotherapy and radiobiology, in particular, for describing cell survival under high-dose radiation^18^.

To overcome some inconsistencies of target theory, the “Linear-Quadratic (LQ) model” was proposed by the dual radiation action theory^19^ and molecular theory^20^. In the LQ model, the cell survival fraction *S* with dose *D* is given by


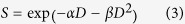


where, the dose coefficients *α* and *β* are the radiosensitivity parameters. Generally, the LQ model is effective in the low-dose range for low linear energy transfer (LET) radiotherapy, but less effective in fitting the dose-response curves at high-dose range, which are widely used on stereotactic body radiotherapy (SBRT)^21^,^22^. In spite of this, the LQ model is still the most commonly used medical theory, which is used to describe the radiation effects for the fractionation schemes at present^22^,^23^,^24^,^25^.

A number of other cell survival models dedicated to high-LET radiation have also been proposed, such as the amorphous track structure model (ATSM)^26^,^27^,^28^ and the more recent Local Effect Model (LEM)^29^,^30^,^31^,^32^. Both models are based on the assumptions of simplified biological targets and radiation fields (amorphous particle track)^33^. The target is assumed to be an infinitely thin disc, its size or cross-section being endpoint dependent. More specifically, it is assumed in the ATSM that a set of cellular subtargets, representing the sensitive site, has been activated or inactivated to induce the effects. Thus, the cross-sectional area is not always equal to the geometrical size of the target. However, there is no concept of subtarget in the LEM, and the response of the biological target (cell nucleus) is assumed to be determined by local dose deposition events. Both models assume that the radiation field is entirely described by its dose distribution due to δ-electrons originating from ionization events, while neglecting the contributions from excitations and nuclear stopping. Under this assumption, there is no LET variation from particles or even stopping particles within the target. Hence, the basic assumptions of the ATSM and the LEM are questioned^33^,^34^. Generally, all these models mentioned above can be ascribed to the “targeted effects models”^8^,^35^. In addition, a series of non-targeted effects models have been developed^36^, while generally speaking, their applications can not be taken into account in radiotherapy due to the limited specific data set fitting^10^.

In fact, the response of different organisms to ionizing radiation depends on the radiation quality and the intrinsic characteristics, which mainly include repair mechanisms of the living organism. Therefore, some authors have proposed a series of repair models from different points of view^4^,^37^,^38^,^39^,^40^. Most of these models could better predict the cellular repair capacity for low LET radiation, while they usually become ineffective for high LET radiation. For example, some researchers regarded the ratio *α*/*β* in the LQ model as an indicator of cellular repair capacity^3^,^41^, while it tends to overestimate the cellular repair capacity for high LET radiation.

In this work, we firstly proposed a simple radiobiological model, which tries to reflect cellular repair effects together with indirect effects of radiation. Then, we discussed the relationships between this model and the target model, as well as the LQ model. After that, we tested this model by fitting the representative and widely used experimental data sets reported in publications. Finally, the present model was used to predict the radiosensitivity and target size of the cell, and calculate the biologically effective dose (BED) for the treatment planning in clinical applications.

## Methods and Materials

### Theoretical Model

When an organism is subject to irradiation, there is no doubt that “the irradiated tissue (cell set) will be affected by radiation” is a deterministic event, and “the target of a cell in this tissue may be hit by radiation particles” is a stochastic event. However, “the cellular response after its target being hit” should be regarded as a fuzzy event, because the response can be irreversible or reversible change in structure and function (Cell death or inactivation is only the most severe damage response.), depending on radiation characteristics and cell types. Both the stochastic event and the fuzzy event are used to express the characteristics of uncertainty, and the difference between them is the former has its precise boundary, whereas the latter boundary is not sharp^42^. Thus, the relationship between “hit” and “inactivation” in traditional target theory should be generalized. The negation operator in fuzzy mathematics can provide a way to implement this idea, which carries the sense of complement^42^.

Let *p*_0_ be the hit probability, the survival fraction *S* in Lea’s target theory is


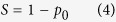


which can be regarded a standard negation operator in interval [0, 1]. This implies that “If the target of a cell is hit, it will certainly lead to the cell inactivation”^18^. If “the cellular response after its target being hit” is a fuzzy event, the standard negation operator defined in equation (4) may be replaced by a more general negation operator,


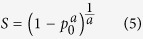


which is one of the popular complement functions, i.e., so-called Yager negation operator^42^,^43^, where *a* is the parameter of negation (*a* > 0). When *a* = 1, it means that one hit necessarily lead to one inactivation, and equation (5) will be reduced to equation (4) in target theory; *a* > 1 means that one hit will lead to less than one inactivation, for the cellular repair mechanisms are likely at work strongly; *a* < 1 implies that one hit will lead to more than one inactivation, because the indirect effects, which are caused by the damage of the radiation-induced free-radical attack to the key target in the cell, are likely to become dominant.

Combining equation (1) and (5), one can obtain the general survival equation, which is called as the generalized multi-hit multi-target (GMHMT) model


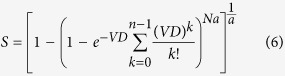


when both *N* and *n* are equal to 1, the GMHMT model can be simplified to


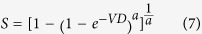


which is called as the generalized single-hit single-target (GSHST) model.

### Low-dose and high-dose approximation

Taking the natural logarithm of both sides of equation (7), one can obtain


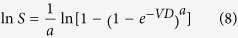


In the low-dose region, 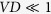. If the logarithmic term in equation (8) is expanded into a Taylor series, neglecting the second and higher order terms, one can get


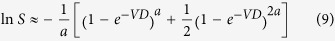


Furthermore, using the linear approximation of Taylor series expansion of exponential function and power function, equation (9) can be reduced to the LQ model


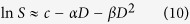


where 
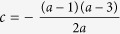
, *α* = (2 − *a*)*V*, and 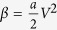. It can be seen that *α*, *β* and their ratio *α*/*β* all depend on the parameter *a* and target size *V*.

On the other hand, in the high-dose region, 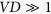. If the power function in logarithmic term in equation (8) is expanded into a Taylor series, again neglecting the second and higher order terms, equation (8) can lead to the same result with the single-hit multi-target model at high-dose


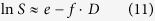


where 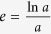,and 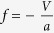. And, the slope of the approximate expression is the reciprocal of the ratio *a*/*V*.

Thus, the GSHST model can be naturally reduced to the LQ model and the multitarget model asymptote at low-dose and high-dose ranges, respectively. To unify the LQ model survival curve for the low-dose range and the multitarget model asymptote for high-dose range, it is not necessary to artificially introduce the piecewise-defined function with a transitional discontinuity in the universal survival curve model^44^,^45^. In addition, some authors attempt to develop integrated theories in radiobiology^45^, for example, Sutherland proposed a repair dependent model with the product of a single exponential and an Euler gamma function, which could accurately describe the survival fraction and the probability of transformation^46^,^47^. In these models, since curve-fitting is the main issue, the model with smallest number of parameters that fits the data well is the best choice^48^. However, for example, there are as many as four parameters in the repair dependent model^46^,^47^.

So far, the ambiguous descriptions of “target” were used in a variety of different ways^7^,^18^. In fact, the detailed concepts of the “target”, which may be the cell nucleus, chromosomes, or DNA, are still open at present^7^. Therefore, the “number of targets in a cell” was introduced artificially in various target theories. The similar case is for the concept of “the number of hits per target”. In fact, both “number of targets in a cell” and “the number of hits per target” are the parameters of phenomenological target theories, they even not be integers^49^,^50^. On the other hand, it was found that different versions of the GMHMT model could give the similar dose-response curves by adjusting the parameter *a*. In this work, we assume that there is only one “effective target” in a cell, and one hit by radiation on the “effective target” could cause varying degrees of cell death, depending on the characteristics of radiation, the cellular repair abilities and indirect effects, etc. Thus, only the GSHST model was used to describe the general dose-response curves in the theory.

## Results and Discussion

### Simulated survival fraction

**Figure 1:**
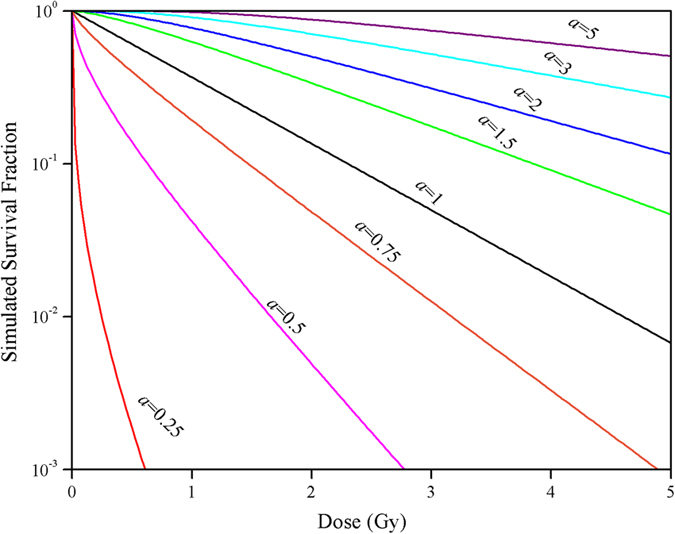
Simulated survival fraction of the present model on a semilogarithmic scale as the parameter *a*, assuming that the target volume *V* = 1.

Figure 1 shows the simulation results of the GSHST model to describe the cell survival, where the parameter *V* takes the value 1 and *a* ranges from 0.25 to 5. When *a* is equal to 1, the GSHST model will be reduced to SHST model in Lea’s target theory. It was shown that the dose-response curves changed with parameter *a* when parameter *V* was fixed: if *a* is greater than 1, the dose-response relationship showed a smooth curve with a larger shoulder at the low dose, which means that the survival fraction decreased slowly with increasing *a* values; on the other hand, if *a* is less than 1, the dose-response relationship showed a steep tendency, which means that the survival fraction decreased rapidly with decreasing *a* values.

### Comparison with experiments

The detailed datasets in the collection of published measurements, and methods of model fitting were listed in Supplementary information (SI). The results of fitting equation (2), (3), and (7) to the cell survival data are shown in Supplementary Fig. S1-S8 (SI), respectively. Regression analysis showed that the GSHST model, the SHST model, and the LQ model all could give significant fits to experimental results (*P* < 0.05) (Supplementary Table S1 and S2, SI).

Overall, the goodness of fit of the GSHST model is better than that of the SHST model, approximate to that of the LQ model (Supplementary Table S1 and S2, SI). In addition, we will see that, the ratio *a*/*V* in the GSHST model is a more effective indicator than the ratio *α*/*β* in the LQ model to reflect the cellular repair capacity.

### RBE and cellular repair capacity

Based on the results of the GSHST model fitting the experimental data, the RBE_37_ (see Supplementary equation (S3) to (S5) in SI for more details) for different LET radiations could be calculated in five cell lines (Supplementary Fig. S9, SI). It can be seen that, in general, the RBE_37_ values firstly increased with LET, after reached a peak at around 150 ~ 200 keV/μm, then decreased with LET. However, for xrs5 mutant cells, the maximum of RBE_37_ was significantly less than that of other cells. It should be pointed out here that, because the experimental data was deficient when LET > 252 keV/μm, there was no significant decline in the higher-LET region for T1 cells. If the corresponding experiments had been done, it is inferred that a significant RBE_37_ decline in the higher-LET region for T1 cells may appear. So, the relationships between RBE and LET are similar for different cell lines. This conclusion is consistent with the previous reports^51^.

**Figure 2:**
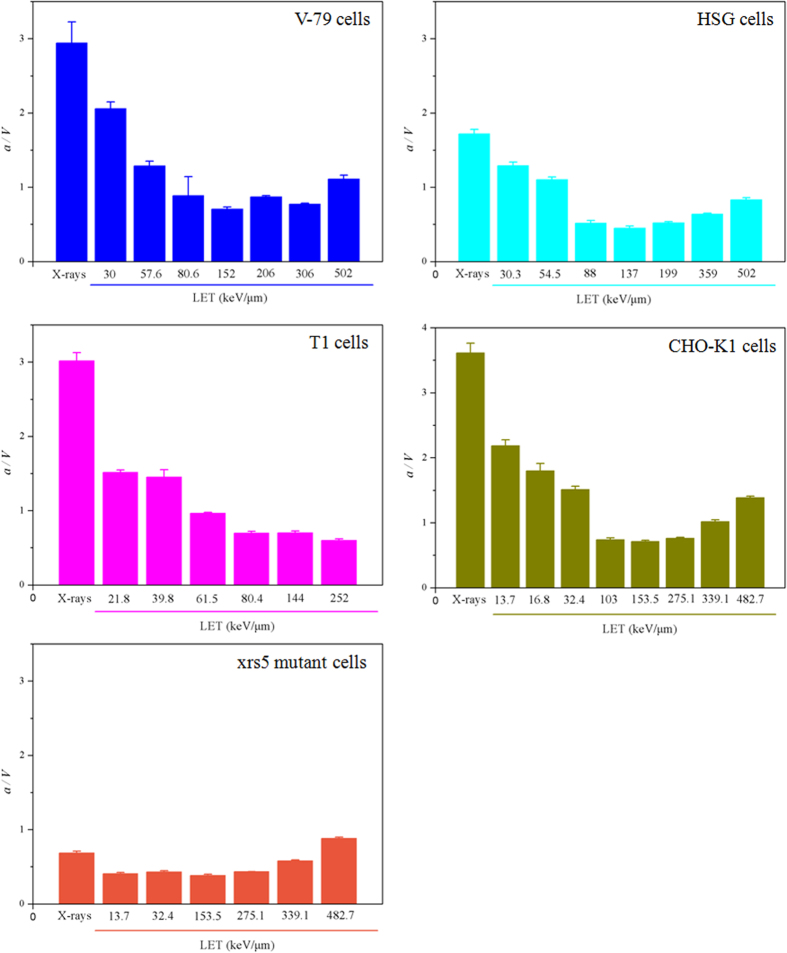
Ratios *a*/*V* (unit: Gy) in five cell lines as a function of LET using the present model for regression analysis. Values represent the arithmetic mean ± standard error.

The ratios *a*/*V* for different LET radiations in the five cell lines could be calculated, the results were listed in Fig. 2. It can be seen that the relationship between *a*/*V* and LET is generally dependent on the cell-line types: for Chinese hamster cells (V-79), human salivary gland tumor cells (HSG), and Chinese hamster ovary cells (CHO-K1), the *a*/*V* decreased with increasing LET and the minimum was attained at around 150 keV/μm, then increased slightly with increasing LET. For the T1 cell, the *a*/*V* gradually decreased with increasing LET. Whereas all of the *a*/*V* are less than 1 in xrs5 mutant, which are obvious lower than that of other cells.

**Figure 3:**
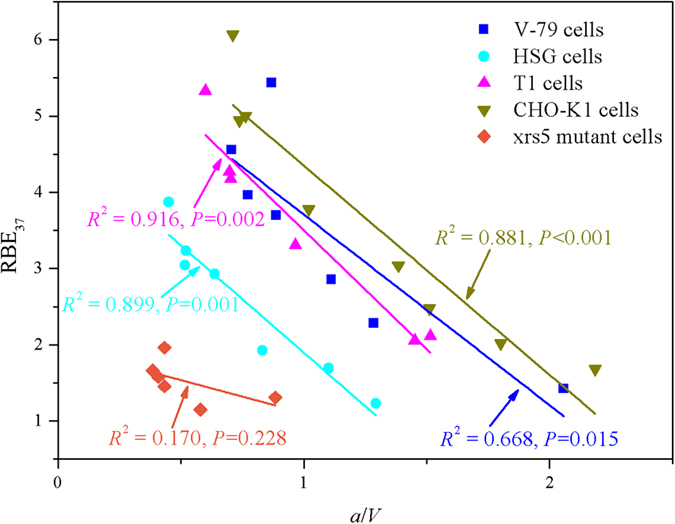
Linear regressions of RBE and the ratios *a*/*V* (unit: Gy) together with the corresponding values in five cell lines irradiated by ^12^C with different LETs in this study.

Combining the discussion in the last two paragraphs, one can see that RBE and *a*/*V* for different cell lines exhibit the opposite trend with increasing LET. As shown in Fig. 3, indeed, the significant linear correlations were observed between RBE_37_ and the ratios *a*/*V* for V-79, HSG, T1, and CHO-K1 cells (*P* < 0.05). However, there was no significant correlation between RBE_37_ and *a*/*V* for xrs5 mutant (Adj. *R*^2^ = 0.170, *P* = 0.228). The absence of Ku80 component makes the xrs5 mutant difficult to recruit DNA-PKcs, which is followed by other repair proteins in the repair pathway of non-homologous end-joining (NHEJ)^52^, and this can result in the lack of cellular repair capacity.

The above analysis shows that the ratio *a*/*V* in the present model could reflect the cellular repair capacity. The ratio decreases with increasing LET, after reaches a nadir at around 150 ~ 200 keV/μm, then increases with LET. The above results also imply that RBE is probably related to the cellular repair capacity. So, the declining of RBE at high LET region (>100 ~ 200 keV/μm) observed in radiobiology experiments may be due to the increasing cellular repair capacity. Previous studies also show that the very end of range (largest LETs) for certain particles (such as helium with LET = 123 keV/μm) can induce less unrepaired damages than those of low LET (such as helium with LET = 40 ~ 81 keV/μm)^53^, which is consistent with the present hypothesis. In other words, the high-LET radiation can activate the repair mechanisms or enhance the cellular repair capacity. So far, the declining effects had been described by several mechanisms^54^, such as “overkill effects”, “thindown”, etc. Each of these speculations considered the saturation mechanism of detriment, while neglected the repair mechanism in organisms. In the case of high LET radiation, more than 90% lesions are associated with very complex damages. Several repair pathways, including NHEJ, homologous recombination, microhomology-mediated end-joining, and alternative nonhomologous end-joining, may sequentially attempt to recover it^55^. The choice of the appropriate pathway is regulated by three major factors, which include the damage complexity, cell cycle phase, and the speed of different repair mechanisms^56^.

**Figure 4:**
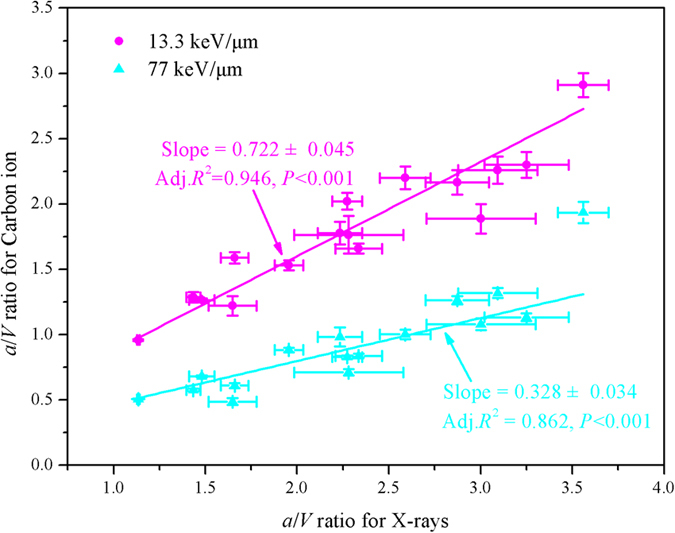
Linear correlations between two sets of the *a*/*V* (unit: Gy) values under X-rays and low LET ^12^C radiation (13.3 keV/μm, pink), X-rays and high LET ^12^C radiation (77 keV/μm, cyan).

It was interesting to find that significant differences of *a*/*V* values under X-rays, low and high LET ^12^C radiations were observed (*P* < 0.05) (Supplementary Fig. S10, SI). In addition, significant linear correlations between two sets of the *a*/*V* values under X-rays and low, and high LET ^12^C radiations were both found (*P* < 0.001) (Fig. 4). In the LQ model, the ratio *α*/*β* can be used as an indicator to reflect the cellular repair capacity. This indicator works well except for the A-172 cell line in the case of low LET ^12^C radiation. However, it does not work in high LET ^12^C radiation^41^. In contrast with these results, the previous results showed that the ratio *a*/*V* in the GSHST model maybe considered a robust indicator to reflect the cellular repair capacity, which is suitable for both low and high LET radiotherapy.

It can be seen that the low LET slope was 2.20 times higher than the high LET slope (Fig. 4), which suggested that the cellular repair capacity was mainly dependent on LET. Furthermore, the above results also indicated that the cellular repair capacity should be a complex function of LET, and there should exist the threshold to strengthen or activate the repair mechanism for different cell lines.

### Cellular repair capacity dependent radiosensitivity

The ratios *a*/*V* for various human cell lines with X-rays, low and high LET ^12^C radiations were shown in Supplementary Fig. S10 (SI). The ratios *a*/*V* fluctuate in various cell lines under the same irradiation conditions, indicating that the cellular repair capacity was also dependent on cell types. Furthermore, the mean RBE_37_ under high LET ^12^C radiation (2.247 ± 0.333) were 1.70-fold higher than those under low LET ^12^C irradiation significantly (*P* < 0.05) (Supplementary Fig. S11, SI).

**Figure 5:**
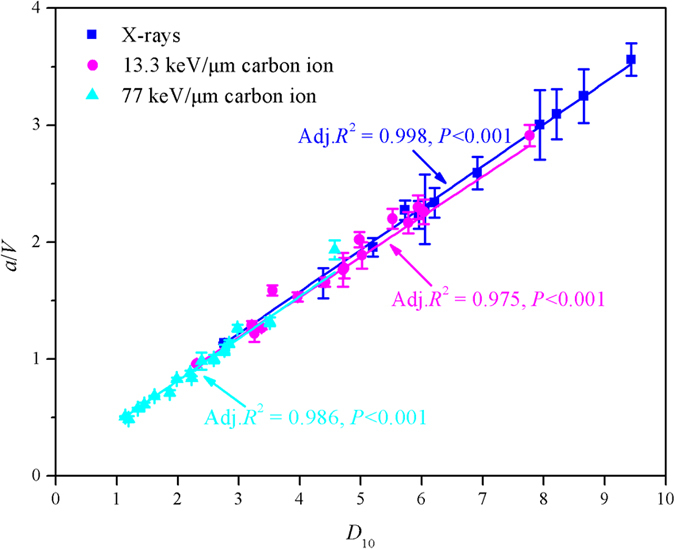
Linear regressions of the ratios *a*/*V* (unit: Gy) and *D*_10_ (unit: Gy) together with the corresponding values in sixteen cell lines under X-rays, low and high LET ^12^C radiations (13.3 and 77 keV/μm) in this study.

The *D*_10_ (dose required for 10% survival) in the present model could be calculated by the following equation


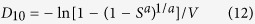


where *S* is equal to 0.1, and *D*_10_ is regarded as a classic indicator for radiosensitivity^41^. By linear regression analysis, it was found that *a*/*V* was linearly correlated well with the *D*_10_ for X-rays, low and high LET ^12^C radiations, respectively (*P* < 0.001) (Fig. 5). These results indicated that the radiosensitivity of a particular cell type was dominated by the cellular repair capacity.

Under X-rays, low and high LET ^12^C radiation conditions, all the maximum and minimum of *a*/*V* appeared in the case of Becker and KS-1 cells. These results imply that the Becker cells has strong radioresistance and the KS-1 cells has strong radiosensitivity, which is consistent with the variation of the RBE_37_ in Supplementary Fig. S11 (SI) and the conclusion coming from Suzuki *et al.*^41^.

### Target size prediction

The size of some biological macromolecules could be calculated by using Lea’s target theory, and the effectiveness of this method had been confirmed^57^,^58^. In the present model, the target size can also be calculated easily. Given the average energy *E*_0_ deposited per “hit” event, the target size (*M*_r_, Da) can be calculated by


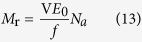


where *f* = 6.2 × 10^15^ eV · g^−1^ · Gy^−1^, is the conversion factor from unit dose to energy deposition events, and *N*_*a*_ is Avogadro’s number. In the present work, we take *E*_0_ = 75 eV, as known empirically from experiment^59^. Supplementary Fig. S12 (SI) depicted that the every target size of sixteen human cell lines has no significant difference under three different treatments (*P* = 0.101). The mean target weight of every sixteen cell lines is (1.12 ± 0.76) × 10^10^ Da, which is in the similar order of magnitude of DNA (the typical mass of a DNA molecule is 3.7 × 10^10^ ~ 1.7 × 10^11^ Da), and obviously bigger than the molecular weights of RNA (approximate to 10^6^ Da) and protein (about 10^4^ Da)^60^. The reason why the predicted values by the present model are less than the typical mass of a DNA molecule is possible associated with the selected average energy *E*_0_ deposited per “hit” event. If *E*_0_ takes a larger value, the estimated target size will be closer to the actual order of magnitude of DNA. The GSHST model indicated that DNA should be the critical and sensitive “effective target” in cell under both low and high LET radiations, which is consistent with the mainstream view that the supposed damage to genomic DNA is the main event of radiation induced^9^,^10^,^18^,^61^. The interaction of radiation with cells can occur through direct interaction of radiation with the “effective target” or through indirect damage caused by elevated radiation - induced ROS production^9^. Nevertheless, the direct and/or indirect effects that depend on radiation quality, will ultimately cause a change that leads to loss of biological activity in the molecule of DNA, and activate different repair mechanisms.

### Calculation and optimization of BED

To show the actual implementation of this model to the treatment planning of carbon ion therapy, we performed a calculation and optimization of BED, which is a characteristic dose value that facilitates comparisons between the effects of different dose-fractionation schemes^44^. The calculation and optimization followed the similar procedures as described by Krämer *et al.*^62^, where BED is defined by the absorbed dose multiplied with the local RBE factors evaluated for a mixed radiation field. Briefly, when applying the present model to therapy planning, the first and most important input data are physical dose distribution profiles *D*(*F*(*x*)), generated by an ensemble of pencil beams at locations *x* with number of particles *F*(*x*) in the water-equivalent material. If the tumor type and size are certain, then the relationships between RBE and *a*/*V*, as well as *a*/*V* and LET can be obtained relatively easy for cultivated cell lines, as shown in Figs. 2 and 3. According to the present model, BED can be got by


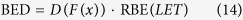


with the physical dose *D* (*F*(*x*)) and RBE (*LET*)


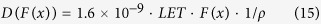



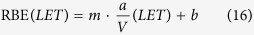


where, as the function of depth, LET can be modulated by a heavy ion beam of energy *E*_beam_^63^,^64^, *ρ* is the density of targeted tissue (or biological material), and the coefficients *m* and *b* can be obtained via the large number of dose-effect curves *in vitro*.

**Figure 6:**
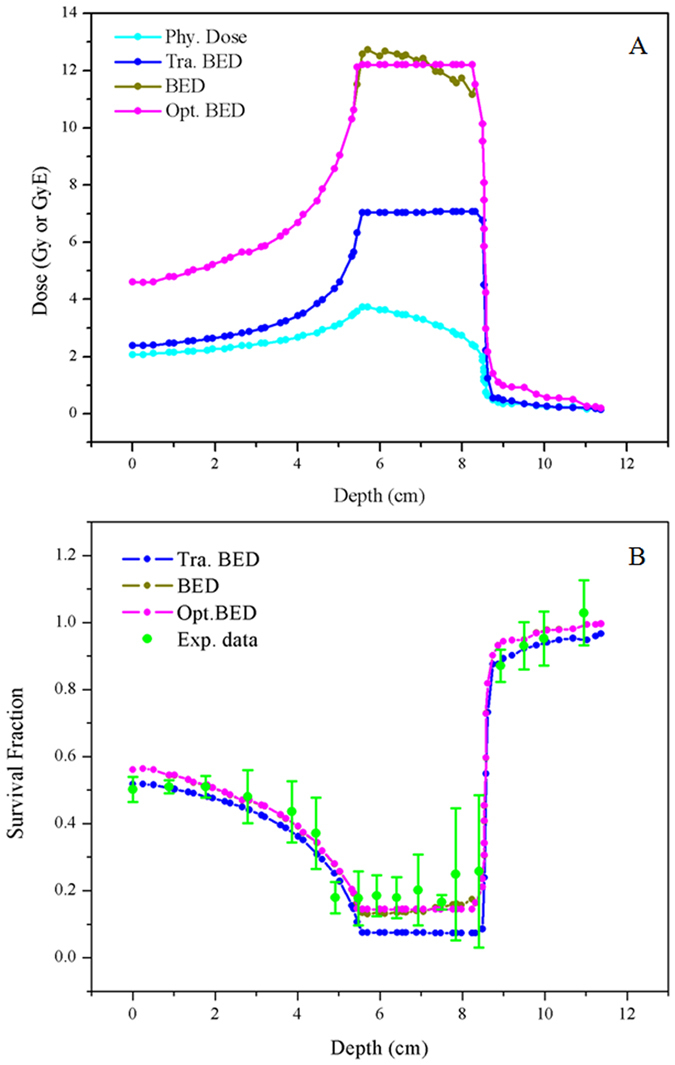
Calculation and optimization of biologically effective dose (BED) (A), and its corresponding cell survival fraction predicted (B) for CHO-K1 cells. Experimental data from reference (59) is adjusted to depth-survival distribution for a 200 MeV u^−1 12^C beam. Phy. dose, Tra. BED, and Opt. BED in the figures represent physical dose, BED calculated by the LQ model and the GSHST model, respectively.

The mean BED are estimated to be 12.2 and 7 GyE at the 3 cm SOBP by depth-dose profile and depth-LET distribution for a 200 MeV u^−1 12^C beam by the LQ and present model, respectively (Fig. 6A). RBE for 37% survival was calculated according to equation (S8) in SI for the LQ model, and equation (16) for the present model, where *m* and *b* could be obtained by the linear regressions of RBE and the ratios *a*/*V* in CHO-k1 cells, as shown in Fig. 3. As an example, Supplementary Fig. S13 in SI showed the comparison of depth RBE distributions obtained directly from the depth LET profiles according to equation (16), where the depth *a*/*V* profiles were derived from the relationship between *a*/*V* and LET in the CHO-K1 cells by linear interpolation.

In order to optimize with respect to BED in the region of SOBP, the same techniques as described by Krämer *et al.*^65^ can be applied, with


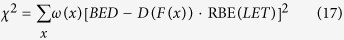



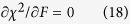


with appropriately chosen weight factors *ω*(*x*) which can be modulated by particles *F*(*x*) at locations *x*, the dose *D* (*F*(*x*)) and RBE (*LET*) are given by equation (15) and (16), respectively. The optimized BED predicted by this method is shown in Fig. 6A. It can be seen that the optimized BED was relatively homogeneous in the region of SOBP, while higher than that of the traditional method, which was in consistent with the clinical practice^66^,^67^. BED was used to predict cell survival fraction by the LQ model, present model, and its optimizing (Fig. 6B), according to the fitted parameters in CHO-K1 cells (Supplementary Table S4 in SI). Comparison with the measured cell survival fraction in experiment shows that the calculated results of the GSHST model is better than that of the LQ model (Supplementary Table S5 in SI).

## Conclusions

In this study, a simple radiobiological model, i.e. GSHST model, is presented. In theory, it overcomes some defects in traditional targeted effects models by considering cellular repair capacity and indirect effects of radiation, and makes the linear quadratic model and multitarget model the low-dose and high-dose limits. In applications, the GSHST model firstly could predict the cell-killing effects accurately and be suitable for various dose and LET ranges, and many cell types. Secondly, the parameter *a*/*V* could well reflect the cellular repair capacity, radiosensitivity, and target size. Thirdly, the present model has a simple mathematical structure for fast computation in radiotherapy, especially the calculation of BED in the treatment planning of clinical applications.

## Additional Information

**How to cite this article**: Zhao, L. *et al.* A generalized target theory and its applications. *Sci. Rep.* **5**, 14568; doi: 10.1038/srep14568 (2015).

## Supplementary Material

Supplementary Information
