# Supplementary material for: A generalized target theory and its applications
Source: Sci Rep. 2015 Sep 28;5:14568. doi: 10.1038/srep14568 (PMC4585963; doi:10.1038/srep14568)
Supplement: Supplementary Information [file srep14568-s1.doc]

**Supplementary information**

**A generalized target theory and its applications**

Lei Zhao^1,2^, Dong Mi^3,*^, Bei Hu^1,2^, & Yeqing Sun^1,2,*^

^1^College of Environmental Science and Engineering, Dalian Maritime University, Dalian, Liaoning, PR China, ^2^Institute of Environmental Systems Biology, Dalian Maritime University, Dalian, Liaoning, PR China, ^3^Department of Physics, Dalian Maritime University, Dalian, Liaoning, PR China

^*^Correspondence and requests for materials should be addressed to Y. S. ([yqsun@dlmu.edu.cn](mailto:yqsun@dlmu.edu.cn)) or D. M. (mid@dlmu.edu.cn). Postal address: NO.1 Linghai Road, Dalian, Liaoning, PR China (116026); Fax: +86-411-8472-5675.

**Data sources**

The available cell survival datasets, including five kinds of cell lines irradiated by ^12^C with different LETs in the range of 13.7 ~ 502 keV/μm and sixteen human cell lines irradiated also by ^12^C with low and high LET values (13.3 and 77 keV/μm were used to represent approximately the low and high LET radiations, which reflects the situations in the Bragg peak and in the entrance channel, respectively), were obtained from the *in vitro* experiments[^1-3^](#_ENREF_1). In addition, as a function of depth, the physical dose and biologically effective dose (BED) profiles of Carbon ion with the initial energy of 200 MeV/u were obtained from reference[^4^](#_ENREF_4). The survival fraction from reference[^5^](#_ENREF_5) were adjusted to depth-survival distribution for a 200 MeV u^-1^ ^12^C beam.

All these radiobiology data were representative and had been widely used in the test of model validity. Chinese hamster cells (V-79), human salivary gland tumor cells (HSG), T1 cells, and Chinese hamster ovary cells (CHO-K1) are the wild type of normal human cell lines, while xrs5 mutant is not. The xrs5 mutant is derived from the CHO-K1 wild-type cell line on the basis of hypersensitivity to X-rays[^6^](#_ENREF_6), which is deficient in DSB repair, due to a lack of the Ku80 component of the active DNA-PK complex[^7^](#_ENREF_7). Ku80 plays a key role in the repair of DSB induced by low LET radiation.

The cell survival datasets with X-rays irradiation were selected as a reference. Because the usual experimental data were reported in the form of figures, the mean survival without error bars, was extracted by Getdata Graph Digitizer (version 2.24). The detailed irradiation parameters, including irradiation type, initial energy, LET, dose rate, and as well as the chosen cells were listed in Table S3.

**Model fitting**

The cell survival data for different cell lines and LET values with selected doses were fitted to the GSHST, STSH, and LQ models (see equation (2), (3), and (7) in the text), respectively. The correlations between the models and the dose-response data were evaluated from the statistical values of significance level (*P*), adjusted squared correlation coefficient (Adj. *R*^2^), standard error (S.E.) of the estimate. Adj. *R*^2^ was determined by

$\text{Adj.}R^{2}=1-\frac{RSS/\left(n-k-1\right)}{TSS/\left(n-1\right)}$ (S1)

, where *RSS* is the [residual](javascript:void(0);) [sum](javascript:void(0);) [of](javascript:void(0);) [squares](javascript:void(0);), *TSS* is the sum of squares of deviations, *n*-*k*-1 represents the degrees of freedom of *RSS*, and *n*-1 represents the degrees of freedom of *TSS*. When evaluating the quality of a fit to a dataset, the weighted sum of squared residuals is calculated. The weighted sum of squared residuals (also known as *χ*^2^) is .

$\chi^{2}=\sum_{i=1}^{N} \left(\frac{y_{i}-f\left(D_{i}\right)}{\omega_{i}}\right)^{2}$ (S2)

where *y*_i_ is the measured survival when a dose *D*_i_ is applied, *f*(*D*_i_) is the value of the theoretical value at dose *D*_i_, *N* is the number of data points and *ω*_i_ is the stander deviation of a set of measurements where *y*_i_ is the mean calue.

Numerical simulations were performed using R software. Statistical analysis, regression analysis, and fitted curves were conducted using ORIGIN 8.0 (OriginLab) software. The parameters *a* and the target volume *V* in equation (7) (see in the text) were obtained by regression analysis; the ratios *a*/*V* (unit : Gy) and RBE were calculated by Microsoft Excel 2007 software. The *a*/*V* values were reported as arithmetic mean ± standard error (S.E.). The inter-group differences were one way analysis of variance (ANOVA) followed by Dunnett's post hoc test using SPSS 17.0 software. Changes were statistically significant if the probability (*P*) was less than 0.05.

**Calculation of RBE_37_** **by the GSHST model**

Based on the GSHST model, RBE_37_ for 37% survival were calculated by

$\text{RBE}_{37}=D_{37}^{X}/D_{37}^{C}$ (S3)

where $D_{37}^{X}$ and $D_{37}^{C}$ represented the doses at 37% survival for X-rays and ^12^C radiations, respectively. Due to $D_{37}^{X}$ is usually the same for a cell type, equation (S3) becomes

$\text{RBE}_{37}\propto1/D_{37}^{C}$ (S4)

,where $D_{37}^{C}$ can be obtained by

$D_{37}^{C}=-\ln\left[1-\left(1-S^{a}\right)^{1/a}\right]/V$ (S5)

,where *S* = 0.37.

**Calculation of RBE by the LQ model**

If the survival fraction expression in equation (3) (see in the text) is rewritten as

$\ln S=-\alpha D-\beta D^{2}$ (S6)

one can easily obtain

$D=\frac{-\alpha+\sqrt{\alpha^{2}-4\beta\ln S}}{2\beta}$ (S7)

where, the coefficients *α* and *β* are the radio-sensitivity parameters, determined by cell inactivation experiments *in vitro*. When applying the LQ model to therapy planning the first and most important input data are the parameters *α*_X_ and *β*_X_ for X-rays, *α*_ion_ and *β*_ion_ for ^12^C radiation. RBE_37_ are obtained by

$RBE_{37}=\frac{\beta_{C}\left(\sqrt{\alpha_{X}^{2}-4\beta_{X}\ln S}-\alpha_{X}\right)}{\beta_{X}\left(\sqrt{\alpha_{C}^{2}-4\beta_{C}\ln S}-\alpha_{C}\right)}$ (S8)

where *S* = 0.37.

**Table S1 The adjusted squared correlation coefficients (Adj. *R*^2^) obtained by fitting to the data from reference (2) and (3) with the SHST model, the LQ model, and the GSHST model.**

| LET  (keV/μm) | V-79 cells | | | LET  (keV/μm) | HSG cells | | | LET  (keV/μm) | T1 cells | | |
| --- | --- | --- | --- | --- | --- | --- | --- | --- | --- | --- | --- |
|  | GSHST | SHST | LQ |  | GSHST | SHST | LQ |  | GSHST | SHST | LQ |
| X-rays | 0.966 | 0.909 | 0.992 | X-rays | 0.995 | 0.991 | 0.998 | X-rays | 0.985 | 0.963 | 0.990 |
| 30 | 0.992 | 0.962 | 0.998 | 30.3 | 0.994 | 0.971 | 0.999 | 21.8 | 0.998 | 0.989 | 0.996 |
| 57.6 | 0.984 | 0.958 | 0.992 | 54.5 | 0.990 | 0.987 | 0.988 | 39.8 | 0.969 | 0.938 | 0.976 |
| 80.6 | 0.993 | 0.988 | 0.992 | 88 | 0.983 | 0.932 | 0.978 | 61.5 | 1 | 0.989 | 0.997 |
| 152 | 0.986 | 0.978 | 0.981 | 137 | 0.954 | 0.943 | 0.941 | 80.4 | 0.990 | 0.968 | 0.988 |
| 206 | 0.997 | 0.994 | 0.995 | 199 | 0.997 | 0.964 | 0.976 | 144 | 0.994 | 0.958 | 0.994 |
| 360 | 0.998 | 0.987 | 0.992 | 359 | 0.994 | 0.992 | 0.991 | 252 | 0.992 | 0.982 | 0.993 |
| 502 | 0.990 | 0.986 | 0.992 | 502 | 0.988 | 0.963 | 0.995 | - | - | - | - |
| - | - | - | - | - | - | - | - | - | - | - | - |
| Minimum | 0.966 | 0.909 | 0.981 | - | 0.954 | 0.932 | 0.941 | - | 0.969 | 0.938 | 0.976 |
| Maximum | 0.998 | 0.994 | 0.998 | - | 0.997 | 0.992 | 0.999 | - | 1 | 0.989 | 0.997 |

**Table S1 (continued)**

| LET  (keV/μm) | CHO-K1 cells | | | LET  (keV/μm) | xrs5 cells | | |
| --- | --- | --- | --- | --- | --- | --- | --- |
|  | GSHST | SHST | LQ |  | GSHST | SHST | LQ |
| X-rays | 0.948 | 0.875 | 0.979 | X-rays | 0.988 | 0.981 | 0.993 |
| 13.7 | 0.961 | 0.932 | 0.98 | 13.7 | 0.976 | 0.962 | 0.982 |
| 16.8 | 0.934 | 0.861 | 0.935 | - | - | - | - |
| 32.4 | 0.975 | 0.957 | 0.976 | 32.4 | 0.981 | 0.964 | 0.99 |
| 103 | 0.98 | 0.953 | 0.984 | - | - | - | - |
| 153.5 | 0.963 | 0.96 | 0.965 | 153.5 | 0.973 | 0.958 | 0.976 |
| 275.1 | 0.997 | 0.974 | 0.986 | 275.1 | 0.998 | 0.998 | 0.998 |
| 339.1 | 0.985 | 0.97 | 0.987 | 339.1 | 0.982 | 0.970 | 0.986 |
| 482.7 | 0.992 | 0.987 | 0.99 | 482.7 | 0.981 | 0.967 | 0.975 |
| Minimum | 0.934 | 0.861 | 0.935 | - | 0.973 | 0.958 | 0.975 |
| Maximum | 0.997 | 0.987 | 0.990 | - | 0.998 | 0.998 | 0.998 |

**Table S2 The adjusted squared correlation coefficient (Adj. *R*^2^) values obtained by fits to data from reference (2) and (3) with the SHST model, the LQ model, and the GSHST model.**

| Cells | X-rays | | | 13.3 keV/μm Carbon ion | | | 77 keV/μm Carbon ion | | |
| --- | --- | --- | --- | --- | --- | --- | --- | --- | --- |
|  | GSHST | SHST | LQ | GSHST | SHST | LQ | GSHST | SHST | LQ |
| NB1RGB | 0.959 | 0.947 | 0.958 | 0.979 | 0.978 | 0.976 | 0.985 | 0.985 | 0.983 |
| HFL-III | 0.994 | 0.982 | 0.992 | 0.98 | 0.976 | 0.983 | 0.996 | 0.996 | 0.995 |
| LC-1 sq | 0.989 | 0.929 | 0.990 | 0.999 | 0.965 | 0.996 | 0.999 | 0.999 | 0.999 |
| A-549 | 0.982 | 0.941 | 0.990 | 0.990 | 0.939 | 0.994 | 0.990 | 0.989 | 0.989 |
| C32TG | 0.983 | 0.975 | 0.987 | 0.973 | 0.968 | 0.976 | 0.993 | 0.992 | 0.991 |
| Marcus | 0.975 | 0.952 | 0.985 | 0.995 | 0.956 | 0.999 | 0.996 | 0.950 | 0.996 |
| U-251MG(KO) | 0.948 | 0.841 | 0.992 | 0.980 | 0.937 | 0.986 | 0.975 | 0.964 | 0.983 |
| SK-MG-1 | 0.979 | 0.929 | 0.993 | 0.981 | 0.974 | 0.989 | 0.987 | 0.975 | 0.992 |
| KNS-89 | 0.981 | 0.916 | 0.999 | 0.987 | 0.946 | 0.997 | 0.943 | 0.942 | 0.936 |
| KS-1 | 0.999 | 0.997 | 0.998 | 0.999 | 0.998 | 0.999 | 0.999 | 0.999 | 0.999 |
| A-172 | 0.975 | 0.932 | 0.994 | 0.977 | 0.944 | 0.993 | 0.957 | 0.954 | 0.951 |
| ONS-76 | 0.912 | 0.836 | 0.947 | 0.960 | 0.910 | 0.983 | 0.996 | 0.952 | 0.989 |
| KNS-60 | 0.967 | 0.903 | 0.991 | 0.981 | 0.970 | 0.991 | 0.984 | 0.979 | 0.990 |
| Becker | 0.993 | 0.934 | 0.990 | 0.993 | 0.945 | 0.990 | 0.959 | 0.959 | 0.959 |
| T98G | 0.968 | 0.899 | 0.986 | 0.985 | 0.949 | 0.992 | 0.995 | 0.965 | 0.997 |
| SF126 | 0.991 | 0.950 | 0.997 | 0.994 | 0.982 | 0.999 | 0.995 | 0.990 | 0.993 |
| Minimum | 0.912 | 0.836 | 0.947 | 0.960 | 0.910 | 0.976 | 0.943 | 0.942 | 0.936 |
| Maximum | 0.999 | 0.997 | 0.999 | 0.999 | 0.998 | 0.999 | 0.999 | 0.999 | 0.999 |

**Table S3** List of the selected data in this article.

| Cells | General characteristics | Irradiation type | Initial energy  (MeV/u) | LET  (keV/μm) | Dose rate  (Gy/min) | References |
| --- | --- | --- | --- | --- | --- | --- |
| NB1RGB | normal human skin fibroblast | X-rays | 200-kV | - | 0.85 | [^1^](#_ENREF_1) |
|  |  | ^12^C | 290 | 13.3 | 1.2 | [^1^](#_ENREF_1) |
|  |  | ^12^C | 290 | 77 | 1.2 | [^1^](#_ENREF_1) |
| HFL-III | normal embryonic lung fibroblast | X-rays | 200-kV | - | 0.85 | [^1^](#_ENREF_1) |
|  |  | ^12^C | 290 | 13.3 | 1.2 | [^1^](#_ENREF_1) |
|  |  | ^12^C | 290 | 77 | 1.2 | [^1^](#_ENREF_1) |
| LC-1 sq | lung, squamous carcinoma | X-rays | 200-kV | - | 0.85 | [^1^](#_ENREF_1) |
|  |  | ^12^C | 290 | 13.3 | 1.2 | [^1^](#_ENREF_1) |
|  |  | ^12^C | 290 | 77 | 1.2 | [^1^](#_ENREF_1) |
| A-549 | lung, adenocarcinoma | X-rays | 200-kV | - | 0.85 | [^1^](#_ENREF_1) |
|  |  | ^12^C | 290 | 13.3 | 1.2 | [^1^](#_ENREF_1) |
|  |  | ^12^C | 290 | 77 | 1.2 | [^1^](#_ENREF_1) |
| C32TG | amelanotic melanoma | X-rays | 200-kV | - | 0.85 | [^1^](#_ENREF_1) |
|  |  | ^12^C | 290 | 13.3 | 1.2 | [^1^](#_ENREF_1) |
|  |  | ^12^C | 290 | 77 | 1.2 | [^1^](#_ENREF_1) |
| Marcus | brain, astrocytoma | X-rays | 200-kV | - | 0.85 | [^1^](#_ENREF_1) |
|  |  | ^12^C | 290 | 13.3 | 1.2 | [^1^](#_ENREF_1) |
|  |  | ^12^C | 290 | 77 | 1.2 | [^1^](#_ENREF_1) |
| U-251MG(KO) | brain, astrocytoma | X-rays | 200-kV | - | 0.85 | [^1^](#_ENREF_1) |
|  |  | ^12^C | 290 | 13.3 | 1.2 | [^1^](#_ENREF_1) |
|  |  | ^12^C | 290 | 77 | 1.2 | [^1^](#_ENREF_1) |
| SK-MG-1 | brain, astrocytoma | X-rays | 200-kV | - | 0.85 | [^1^](#_ENREF_1) |
|  |  | ^12^C | 290 | 13.3 | 1.2 | [^1^](#_ENREF_1) |
|  |  | ^12^C | 290 | 77 | 1.2 | [^1^](#_ENREF_1) |
| KNS-89 | brain, gliosarcorma | X-rays | 200-kV | - | 0.85 | [^1^](#_ENREF_1) |
|  |  | ^12^C | 290 | 13.3 | 1.2 | [^1^](#_ENREF_1) |
|  |  | ^12^C | 290 | 77 | 1.2 | [^1^](#_ENREF_1) |
| KS-1 | brain, glioblastoma | X-rays | 200-kV | - | 0.85 | [^1^](#_ENREF_1) |
|  |  | ^12^C | 290 | 13.3 | 1.2 | [^1^](#_ENREF_1) |
|  |  | ^12^C | 290 | 77 | 1.2 | [^1^](#_ENREF_1) |
| A-172 | brain, glioblastoma | X-rays | 200-kV | - | 0.85 | [^1^](#_ENREF_1) |
|  |  | ^12^C | 290 | 13.3 | 1.2 | [^1^](#_ENREF_1) |
|  |  | ^12^C | 290 | 77 | 1.2 | [^1^](#_ENREF_1) |
| ONS-76 | brain, medulloblastoma | X-rays | 200-kV | - | 0.85 | [^1^](#_ENREF_1) |
|  |  | ^12^C | 290 | 13.3 | 1.2 | [^1^](#_ENREF_1) |
|  |  | ^12^C | 290 | 77 | 1.2 | [^1^](#_ENREF_1) |
| KNS-60 | brain, giloma | X-rays | 200-kV | - | 0.85 | [^1^](#_ENREF_1) |
|  |  | ^12^C | 290 | 13.3 | 1.2 | [^1^](#_ENREF_1) |
|  |  | ^12^C | 290 | 77 | 1.2 | [^1^](#_ENREF_1) |
| Becker | brain, astrocytoma | X-rays | 200-kV | - | 0.85 | [^1^](#_ENREF_1) |
|  |  | ^12^C | 290 | 13.3 | 1.2 | [^1^](#_ENREF_1) |
|  |  | ^12^C | 290 | 77 | 1.2 | [^1^](#_ENREF_1) |
| T98G | brain, gliblastoma | X-rays | 200-kV | - | 0.85 | [^1^](#_ENREF_1) |
|  |  | ^12^C | 290 | 13.3 | 1.2 | [^1^](#_ENREF_1) |
|  |  | ^12^C | 290 | 77 | 1.2 | [^1^](#_ENREF_1) |
| SF126 | brain, astrocytoma | X-rays | 200-kV | - | 0.85 | [^1^](#_ENREF_1) |
|  |  | ^12^C | 290 | 13.3 | 1.2 | [^1^](#_ENREF_1) |
|  |  | ^12^C | 290 | 77 | 1.2 | [^1^](#_ENREF_1) |
| V-79 | Chinese hamster cells | X-rays | 200-kV | - | - | [^2^](#_ENREF_2) |
|  |  | ^12^C | - | 30 | - | [^2^](#_ENREF_2) |
|  |  | ^12^C | - | 57.6 | - | [^2^](#_ENREF_2) |
|  |  | ^12^C | - | 80.6 | - | [^2^](#_ENREF_2) |
|  |  | ^12^C | - | 152 | - | [^2^](#_ENREF_2) |
|  |  | ^12^C | - | 206 | - | [^2^](#_ENREF_2) |
|  |  | ^12^C | - | 360 | - | [^2^](#_ENREF_2) |
|  |  | ^12^C | - | 502 | - | [^2^](#_ENREF_2) |
| HSG | human salivary gland tumor | X-rays | 200-kV | - | - | [^2^](#_ENREF_2) |
|  |  | ^12^C | - | 30.3 | - | [^2^](#_ENREF_2) |
|  |  | ^12^C | - | 54.5 | - | [^2^](#_ENREF_2) |
|  |  | ^12^C | - | 88 | - | [^2^](#_ENREF_2) |
|  |  | ^12^C | - | 137 | - | [^2^](#_ENREF_2) |
|  |  | ^12^C | - | 199 | - | [^2^](#_ENREF_2) |
|  |  | ^12^C | - | 359 | - | [^2^](#_ENREF_2) |
|  |  | ^12^C | - | 502 | - | [^2^](#_ENREF_2) |
| T1 |  | X-rays | 200-kV | - | - | [^2^](#_ENREF_2) |
|  |  | ^12^C | - | 21.8 | - | [^2^](#_ENREF_2) |
|  |  | ^12^C | - | 39.8 | - | [^2^](#_ENREF_2) |
|  |  | ^12^C | - | 61.5 | - | [^2^](#_ENREF_2) |
|  |  | ^12^C | - | 80.4 | - | [^2^](#_ENREF_2) |
|  |  | ^12^C | - | 144 | - | [^2^](#_ENREF_2) |
|  |  | ^12^C | - | 252 | - | [^2^](#_ENREF_2) |
| CHO-K1 | Chinese hamster ovary cells | X-ray | 250-kV |  | - | [^3^](#_ENREF_3) |
|  |  | ^12^C | 266.4 | 13.7 |  | [^3^](#_ENREF_3) |
|  |  | ^12^C | 197.7 | 16.8 | - | [^3^](#_ENREF_3) |
|  |  | ^12^C | 76.9 | 32.4 | - | [^3^](#_ENREF_3) |
|  |  | ^12^C | 18.0 | 103.0 | - | [^3^](#_ENREF_3) |
|  |  | ^12^C | 11.0 | 153.5 | - | [^3^](#_ENREF_3) |
|  |  | ^12^C | 5.4 | 275.1 | - | [^3^](#_ENREF_3) |
|  |  | ^12^C | 4.2 | 339.1 | - | [^3^](#_ENREF_3) |
|  |  | ^12^C | 2.4 | 482.7 | - | [^3^](#_ENREF_3) |
| xrs5 cells | a mutant derived from the CHO-K1 wild-type cell line on the basis of hypersensitivity to X-rays | X-ray | 250-kV | - | - | [^3^](#_ENREF_3) |
|  |  | ^12^C | 266.4 | 13.7 | - | [^3^](#_ENREF_3) |
|  |  | ^12^C | 76.9 | 32.4 | - | [^3^](#_ENREF_3) |
|  |  | ^12^C | 11.0 | 153.5 | - | [^3^](#_ENREF_3) |
|  |  | ^12^C | 5.4 | 275.1 | - | [^3^](#_ENREF_3) |
|  |  | ^12^C | 4.2 | 339.1 | - | [^3^](#_ENREF_3) |
|  |  | ^12^C | 2.4 | 482.7 | - | [^3^](#_ENREF_3) |

**Table S4** Parameters of the radiobiological model for depth-survival calculation in the equivalent water of CHO-K1 cells.

| Models | *α* | *β* | References |
| --- | --- | --- | --- |
| LQ model | 0.228 | 0.02 | [^3^](#_ENREF_3) |
|  | *V* | *a* |  |
| GSHST model | 0.336 | 1.812 | In this study |
| Optimized methods | 0.334 | 1.802 | In this study |

**Table S5** *χ*^2^-tests of the LQ model and the GSHST model for CHO-K1 cells at a significance level of 0.05.

| Models | *χ*^2^ | *χ*^2^/df | *P* | Hypothesis test (95% confidence) |
| --- | --- | --- | --- | --- |
| LQ model | 32.197 | 1.789 | 0.006 | *H*_0_: rejected |
| GSHST model | 14.586 | 0.810 | 0.482 | *H*_0_: not rejected |


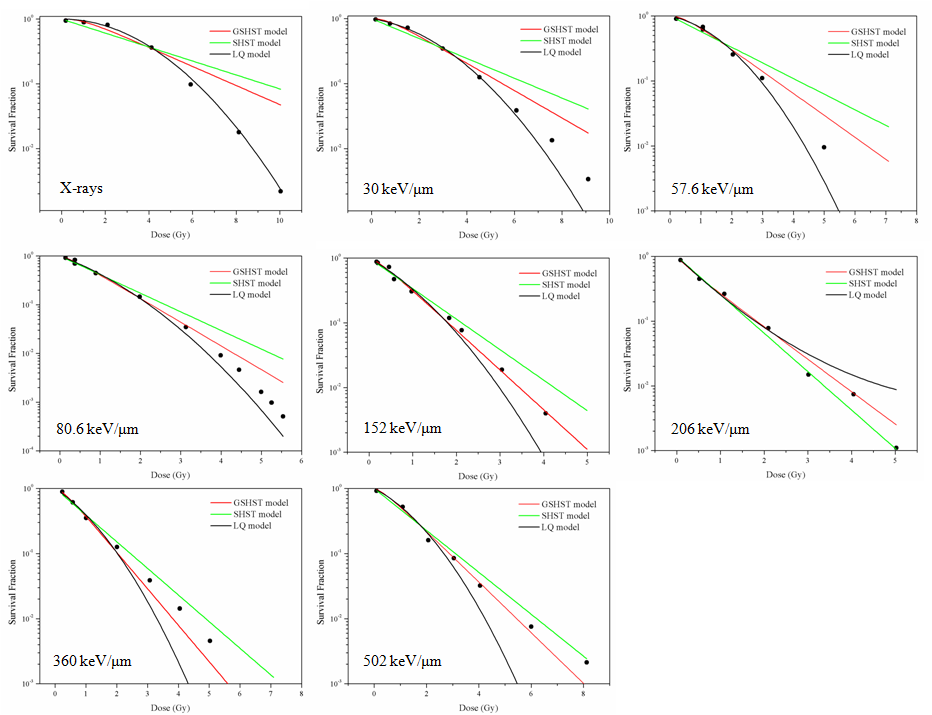


**Figure S1** Survival curves for V-79 cells irradiated with X-rays and ^12^C for seven different LETs as resulting from the simultaneous fit of the whole set of experimental data with the SHST model (green curves), the LQ model (blue curves), and the GSHST model (red curves). Experimental data, shown as scattered points, come from the work of Furusawa et al. (2000).


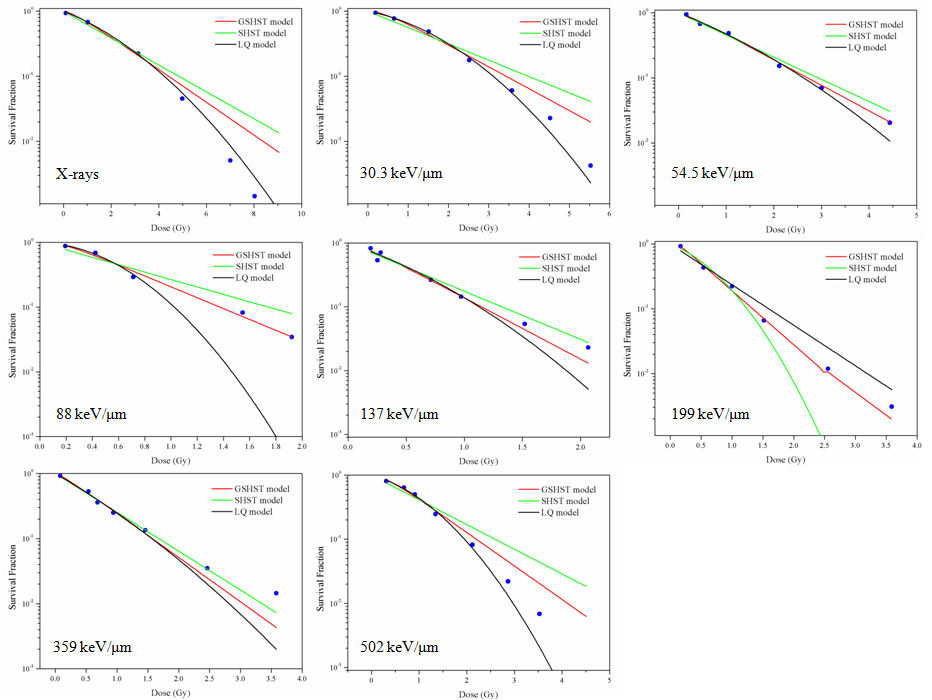


**Figure S2** Survival curves for HSG cells irradiated with X-rays and ^12^C for seven different LETs as resulting from the simultaneous fit of the whole set of experimental data with the SHST model (green curves), the LQ model (blue curves), and the GSHST model (red curves). Experimental data, shown as scattered points, come from the work of Furusawa et al. (2000).


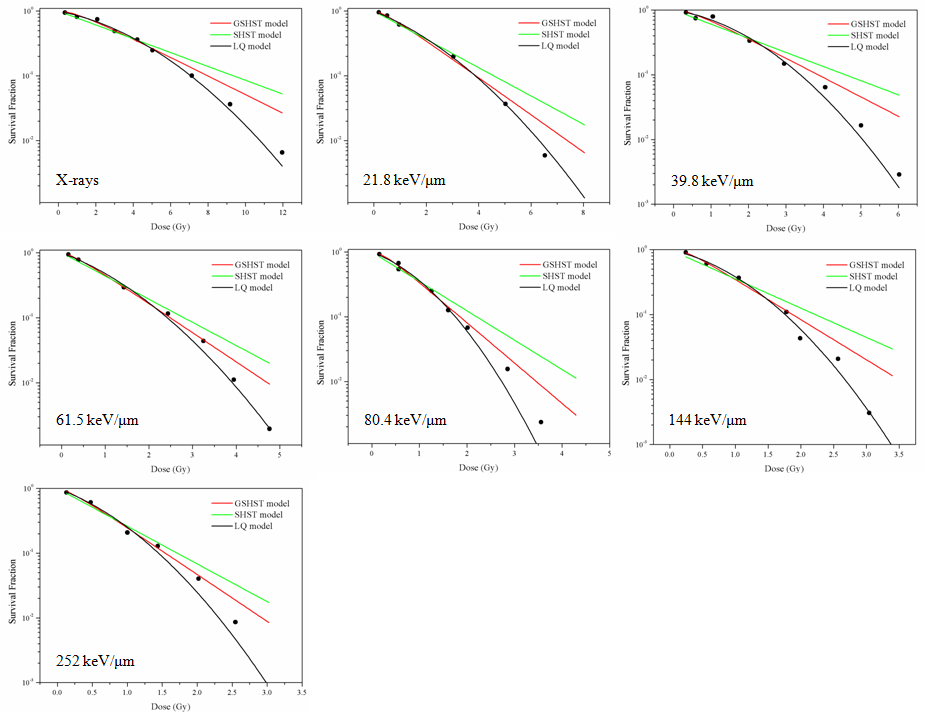


**Figure S3** Survival curves for T1 cells irradiated with X-rays and ^12^C for seven different LETs as resulting from the simultaneous fit of the whole set of experimental data with the SHST model (green curves), the LQ model (blue curves), and the GSHST model (red curves). Experimental data, shown as scattered points, come from the work of Furusawa et al. (2000).


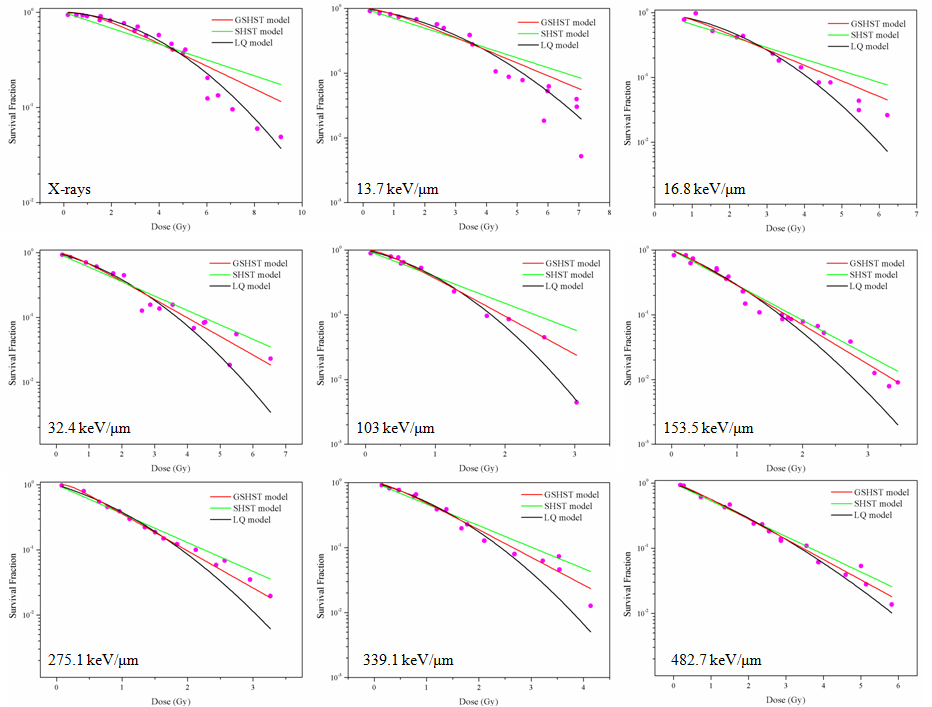


**Figure S4** Survival curves for CHO-K1 cells irradiated with X-rays and ^12^C for seven different LETs as resulting from the simultaneous fit of the whole set of experimental data with the SHST model (green curves), the LQ model (blue curves), and the GSHST model (red curves). Experimental data, shown as scattered points, come from the work of Weyrather et al. (1999).


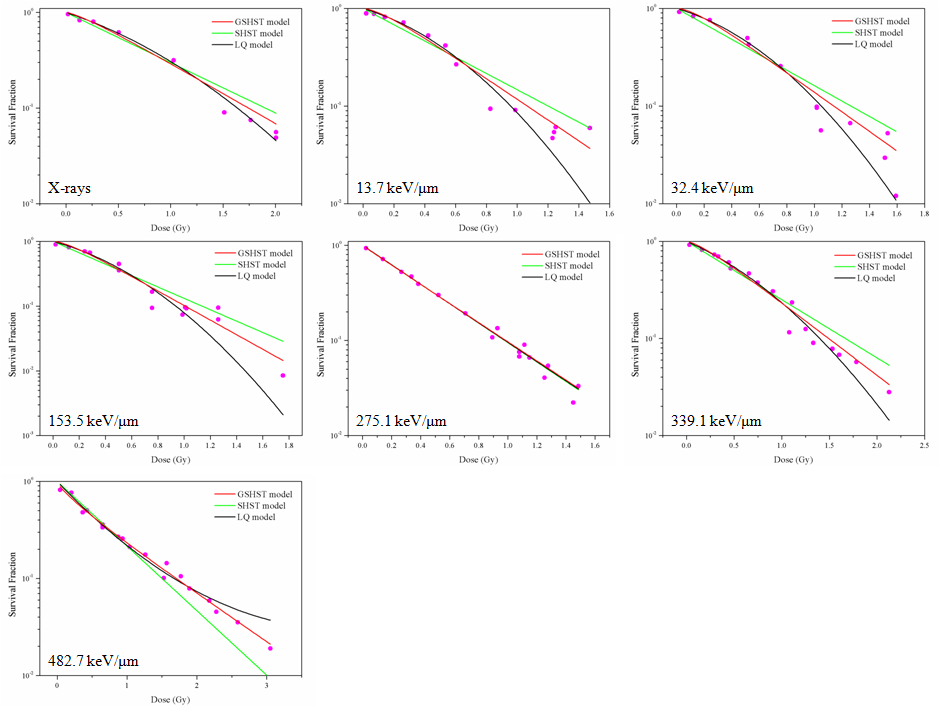


**Figure S5** Survival curves for xrs5 cells irradiated with X-rays and ^12^C for seven different LETs as resulting from the simultaneous fit of the whole set of experimental data with the SHST model (green curves), the LQ model (blue curves), and the GSHST model (red curves). Experimental data, shown as scattered points, come from the work of Weyrather et al. (1999).


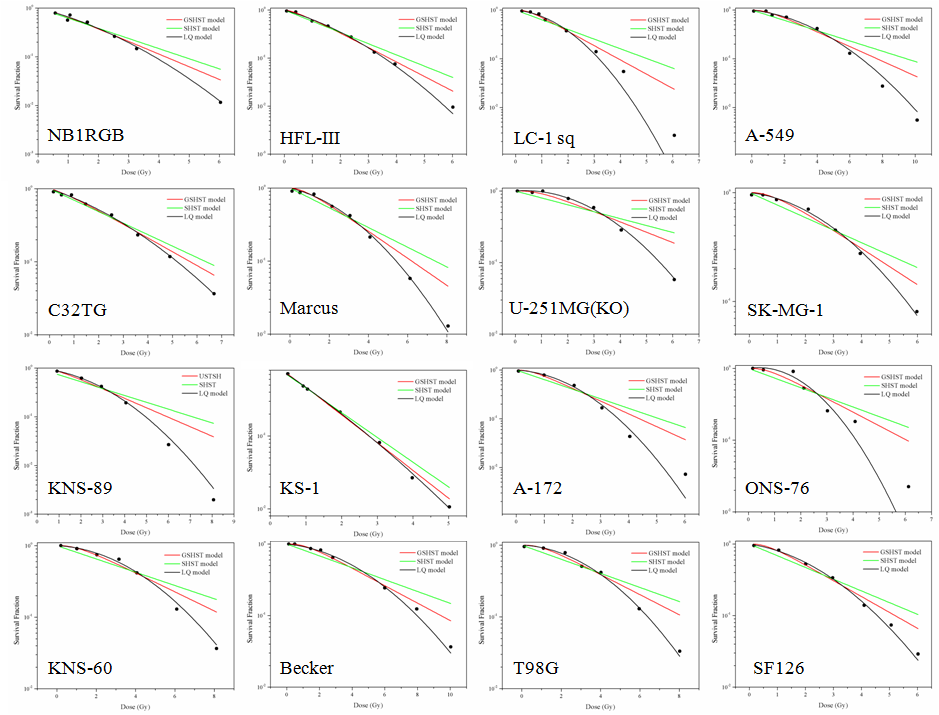


**Figure S6** Survival curves for sixteen cells lines irradiated with X-rays as resulting from the simultaneous fit of the whole set of experimental data with the SHST model (green curves), the LQ model (blue curves), and the GSHST model (red curves). Experimental data, shown as scattered points, come from the work of Suzuki et al. (2000).


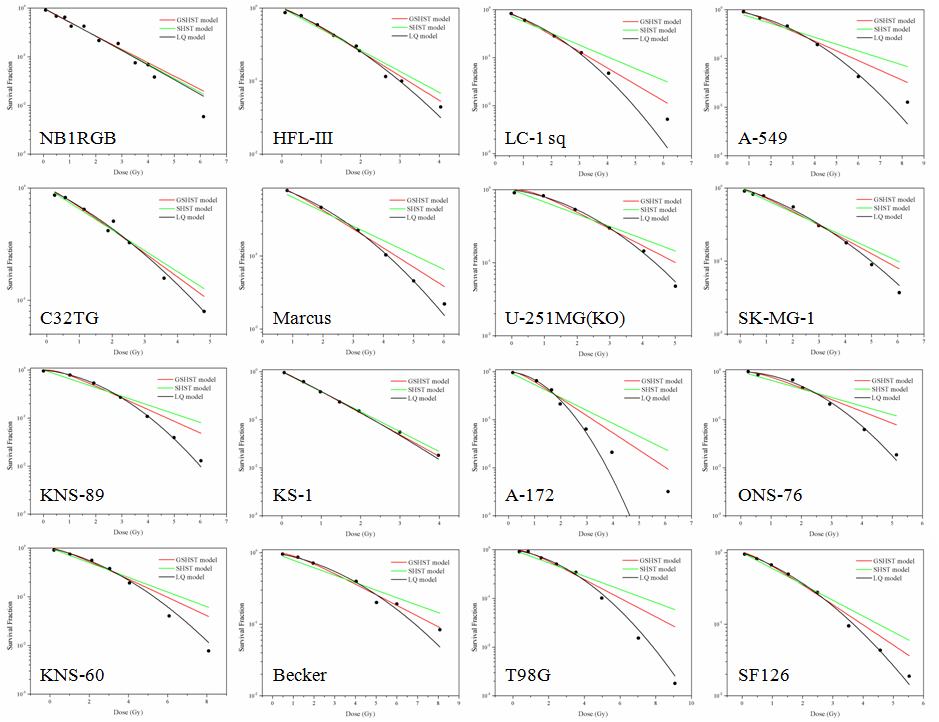


**Figure S7** Survival curves for sixteen cells lines irradiated with low LET (Approximation for 13.3 keV/μm) ^12^C irradiation as resulting from the simultaneous fit of the whole set of experimental data with the SHST model (green curves), the LQ model (blue curves), and the GSHST model (red curves). Experimental data, shown as scattered points, come from the work of Suzuki et al. (2000).


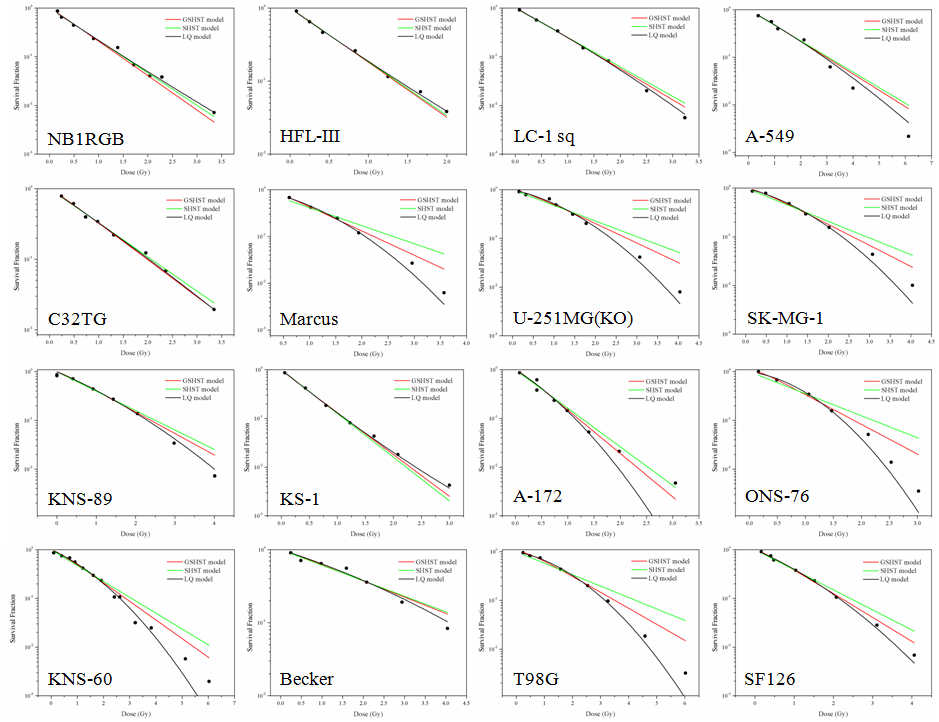


**Figure S8** Survival curves for sixteen cells lines irradiated with high LET (Approximation for 77 keV/μm) ^12^C irradiation as resulting from the simultaneous fit of the whole set of experimental data with the SHST model (green curves), the LQ model (blue curves), and the GSHST model (red curves). Experimental data, shown as scattered points, come from the work of Suzuki et al. (2000).


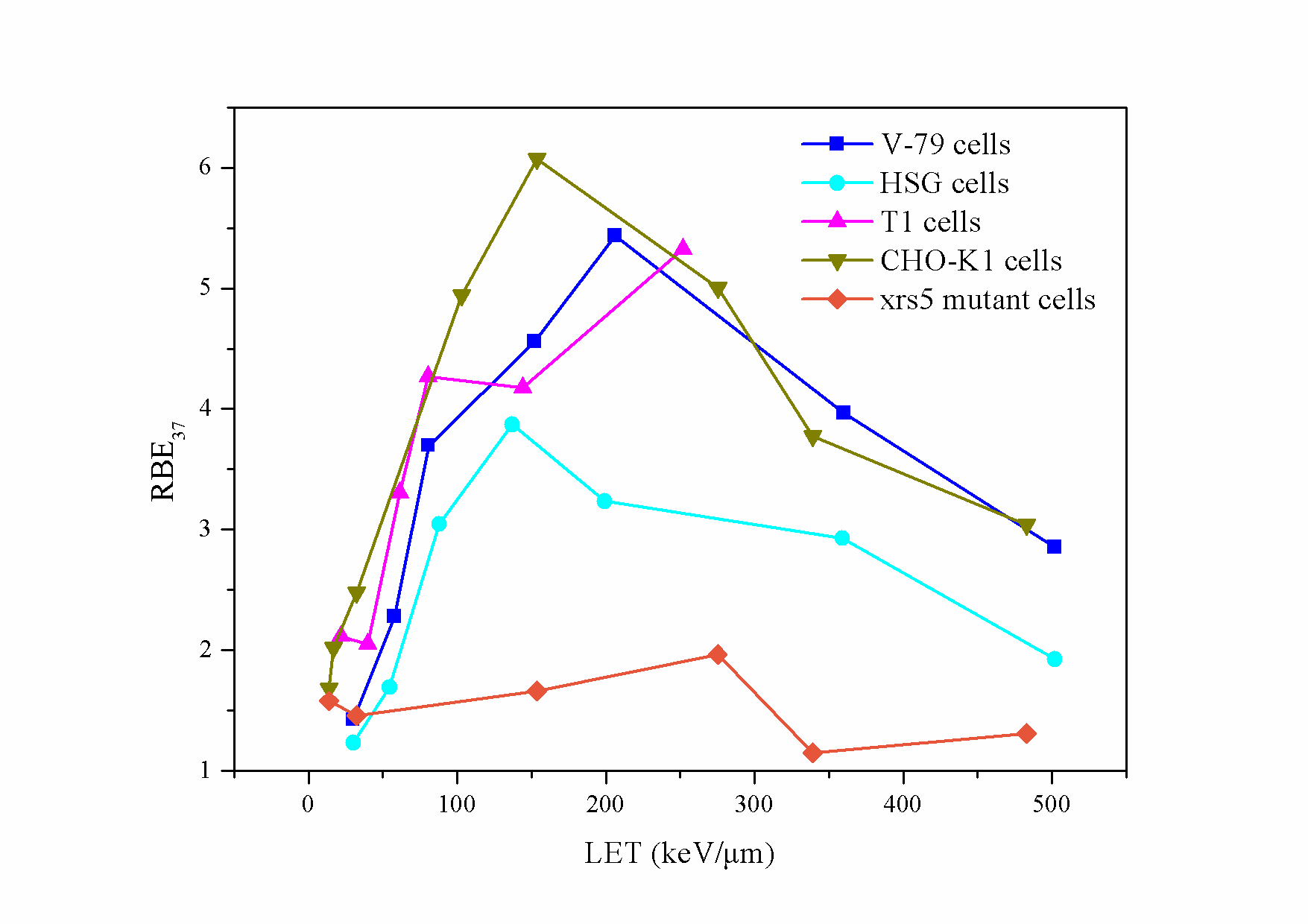


**Figure S9** Relationship between RBE_37_ and LET for V-79, HSG, T1, CHO-K1, and xrs5 cells based on the GSHST model.


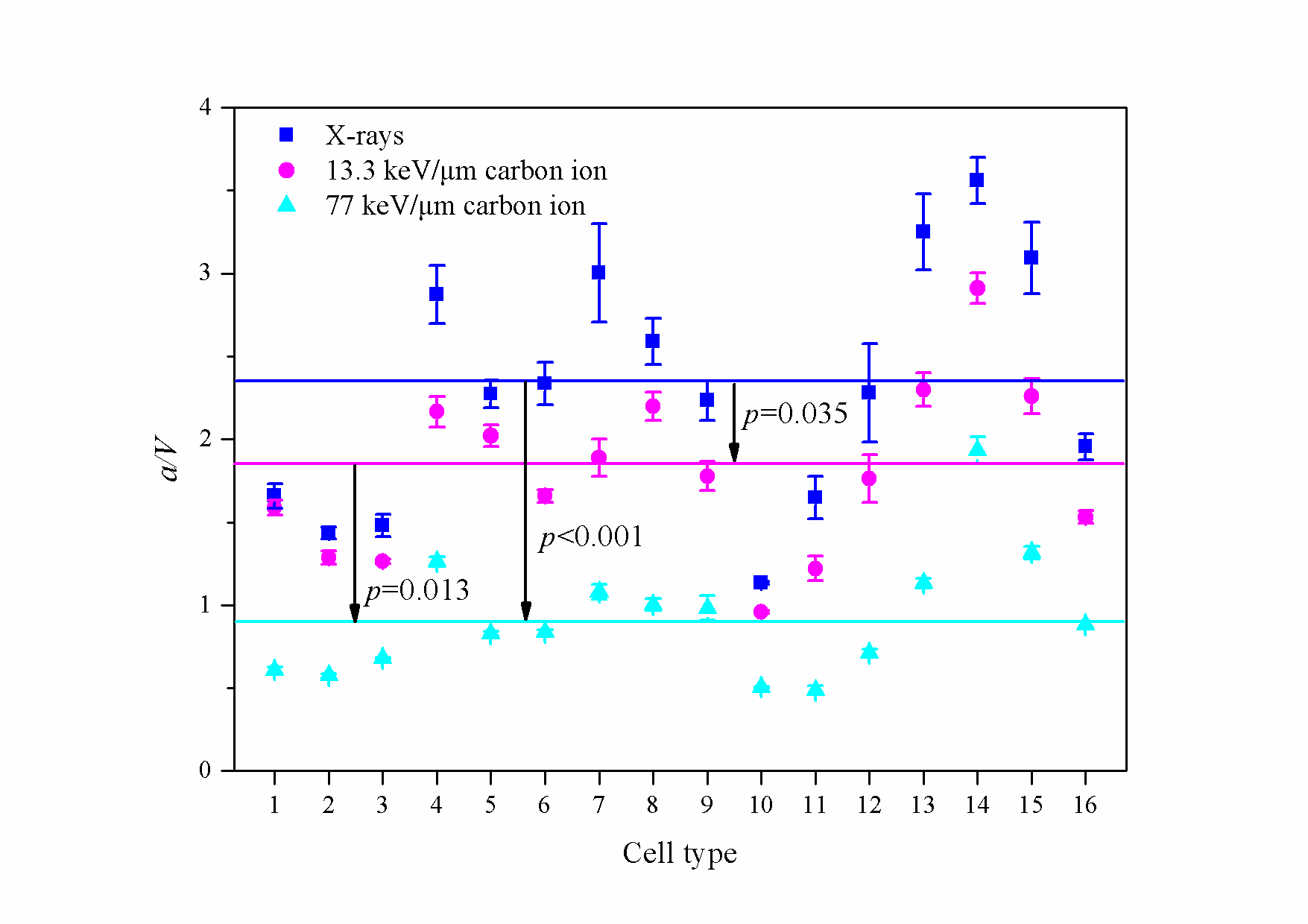


**Figure S10** Ratios *a*/*V* (unit: Gy) in sixteen cell lines irradiated by X-rays and ^12^C with low and high LETs (13.3 and 77 keV/μm). The indicated values represent the arithmetic mean ± standard error. Thereinto, the numbers (1 to 16) represent human cell lines of NB1RGB, HFL-III, LC-1 sq, A-549, C32TG, Marcus, U-251MG(KO), SK-MG-1, KNS-89, KS-1, A-172, ONS-76, KNS-60, Becker, T98G, and SF126, respectively. The detailed characteristics of cell lines are shown in Supplementary Table S3.


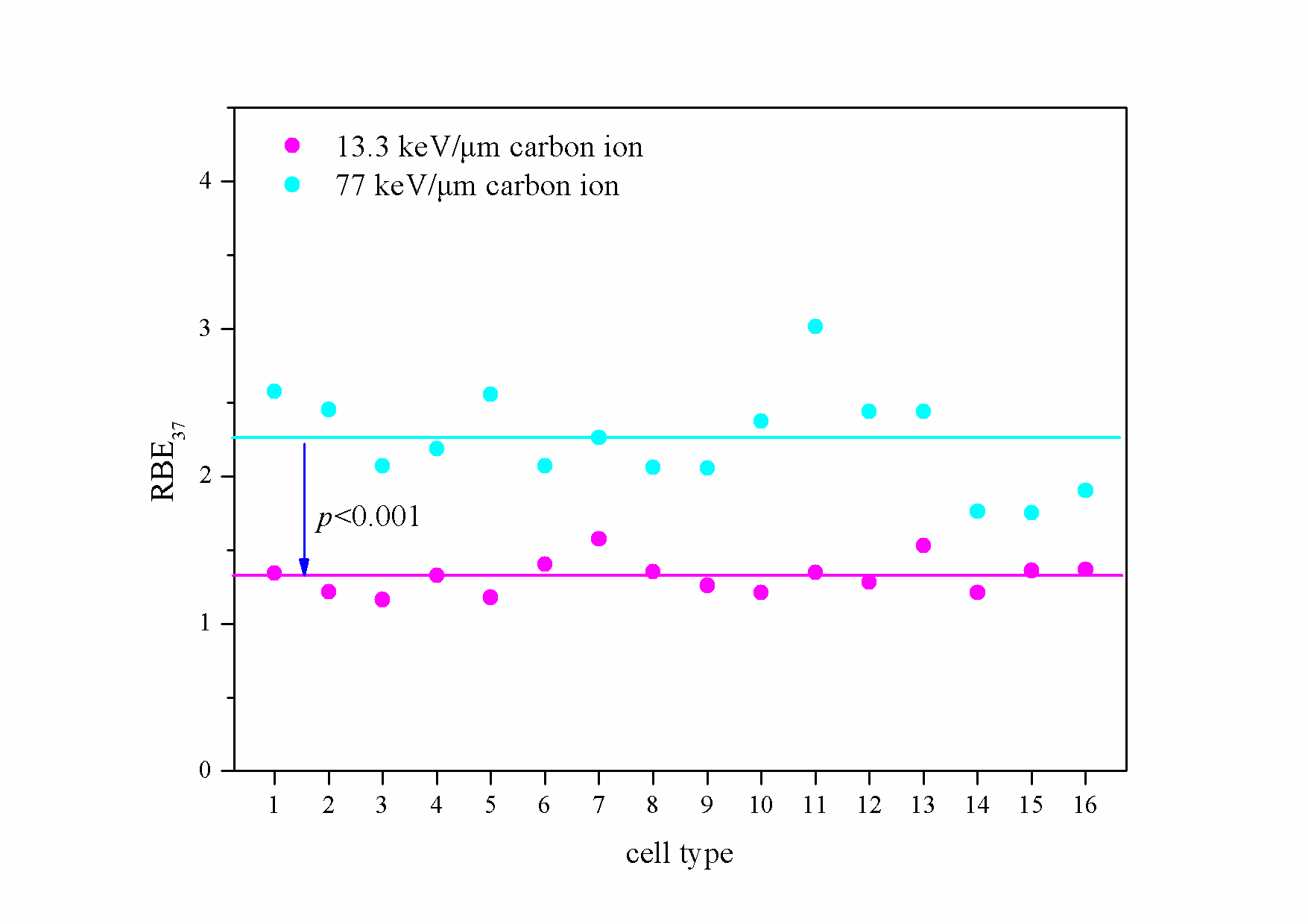


**Figure S11** Relationship between RRE_37_ and LET for sixteen cell lines irradiated by ^12^C with low LET (13.3 keV/μm) and high LET (77 keV/μm) based on the GSHST model. Thereinto, the numbers (1 to 16) represent human cell lines of NB1RGB, HFL-III, LC-1 sq, A-549, C32TG, Marcus, U-251MG(KO), SK-MG-1, KNS-89, KS-1, A-172, ONS-76, KNS-60, Becker, T98G, and SF126. The detailed characteristics of cell lines are shown in Supplementary Table S3.


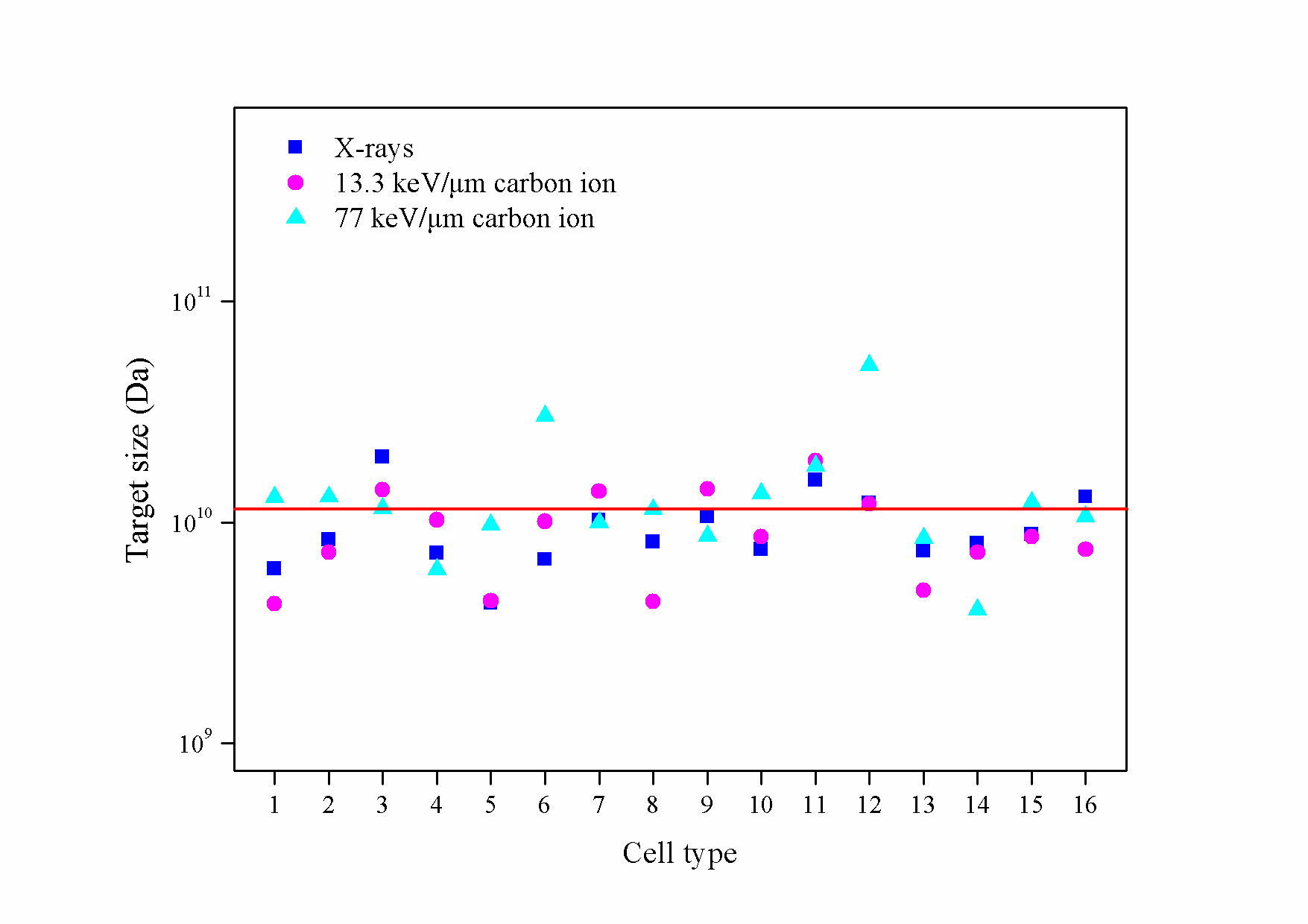


**Figure S12** The estimated target size (unit: Da) for sixteen cell lines under X-rays, low LET (13.3 keV/μm) ^12^C radiations, and high LET ^12^C (77 keV/μm) radiations based on the GSHST model.


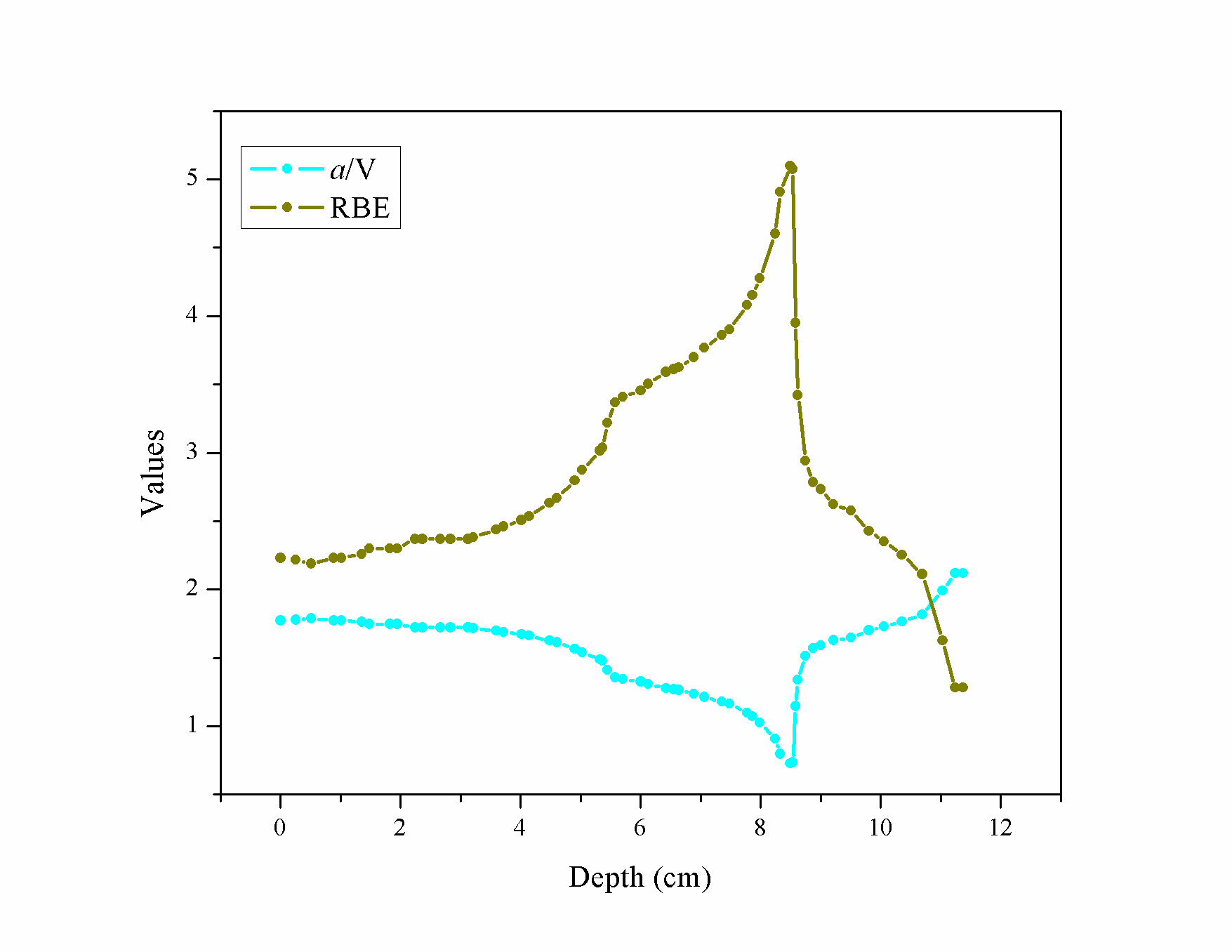


**Figure S13** The ratio *a*/*V* and RBE calculated at each depth in water by the GSHST model.

**References**

1 Suzuki, M., Kase, Y., Yamaguchi, H., Kanai, T. & Ando, K. Relative biological effectiveness for cell-killing effect on various human cell lines irradiated with heavy-ion medical accelerator in Chiba (HIMAC) carbon-ion beams. *Int J Radiat Oncol Biol Phys* **48**, 241-250 (2000).

2 Furusawa, Y. *et al.* Inactivation of aerobic and hypoxic cells from three different cell lines by accelerated He-3-,C-12- and Ne-20-ion beams. *Radiat Res* **154**, 485-496 (2000).

3 Weyrather, W. K., Ritter, S., Scholz, M. & Kraft, G. RBE for carbon track-segment irradiation in cell lines of differing repair capacity. *Int J Radiat Biol* **75**, 1357-1364 (1999).

4 Krämer, M. & Scholz, M. Treatment planning for heavy-ion radiotherapy: calculation and optimization of biologically effective dose. *Phys Med Biol* **45**, 3319 (2000).

5 Mitaroff, A., Kraft-Weyrather, W., Geiß, O. B. & Kraft, G. Biological verification of heavy ion treatment planning. *Radiat Environ Biophys* **37**, 47-51 (1998).

6 Jeggo, P. A. & Kemp, L. M. X-ray-sensitive mutants of Chinese hamster ovary cell line. Isolation and cross-sensitivity to other DNA-damaging agents. *Mutat Res* **112**, 313-327 (1983).

7 Taccioli, G. E. *et al.* Ku80: product of the XRCC5 gene and its role in DNA repair and V(D)J recombination. *Science* **265**, 1442-1445 (1994).
